# Integrated Analysis of Immune-Related circRNA-miRNA-mRNA Regulatory Network in Ischemic Stroke

**DOI:** 10.3389/fneur.2022.889855

**Published:** 2022-06-15

**Authors:** Si Cao, Youjie Zeng, Minghua Chen, Wen Ouyang

**Affiliations:** Department of Anesthesiology, Third Xiangya Hospital, Central South University, Changsha, China

**Keywords:** ischemic stroke, biomarker, bioinformatics, ceRNA, circRNA, differentially expressed genes

## Abstract

**Background:**

Stroke is the leading cause of death and disability worldwide, with ischemic stroke (IS) being the most prevalent type. Circular RNAs (circRNAs) are involved in the pathological process of IS and are promising biomarkers for the diagnosis of IS. However, studies focusing on circRNAs acting as microRNAs (miRNAs) sponges in regulating mRNA expression are currently scarce.

**Methods:**

In this study, expression profiles of circRNAs (GSE195442), miRNAs (GSE117064), and mRNAs (GSE58294) from the Gene Expression Omnibus (GEO) database were analyzed. Differentially expressed circRNAs (DEcircRNAs), differentially expressed miRNAs (DEmiRNAs), and differentially expressed mRNAs (DEmRNAs) were identified by R software. The target miRNAs and target genes were predicted by several bioinformatics methods. Then, we performed Gene Ontology (GO) and Kyoto Encyclopedia of Genes and Genomes (KEGG) pathway enrichment analysis of the DEmRNAs. Subsequently, the protein-protein interaction (PPI) network and the competing endogenous RNA (ceRNA) regulatory network were visualized by Cytoscape software. Finally, we further constructed an immune-related circRNA-miRNA-mRNA regulatory sub-network in IS.

**Results:**

A total of 35 DEcircRNAs, 141 DEmiRNAs, and 356 DEmRNAs were identified. By comprehensive analysis of bioinformatics methods, we constructed a circRNA-miRNA-mRNA regulatory network, including 15 DEcircRNAs, eight DEmiRNAs, and 39 DEmRNAs. FGF9 was identified as an immune-related hub gene. Immune cell analysis indicated a significantly higher level of neutrophils in IS, and the expression of FGF9 was significantly negatively correlated with the level of neutrophils. Eventually, miR-767-5p was predicted as the upstream molecules of FGF9, and circ_0127785 and circ_0075008 were predicted as the upstream circRNAs of miR-767-5p.

**Conclusion:**

Our study provides novel insights into the molecular mechanisms governing the progression of IS from the perspective of immune-related ceRNA networks.

## Introduction

Stroke is the primary cause of mortality and disability worldwide (1). Although the stroke mortality rate has declined in recent years, the prevalence has increased due to population growth and aging (2). It is a sudden disruption of cerebrovascular circulation and includes two subtypes: ischemic stroke (IS) and hemorrhagic stroke (3). Ischemic strokes are more common, accounting for ~87% of all strokes (4). The most effective treatment for IS is intravenous thrombolysis using recombinant tissue plasminogen activator (rTPA). However, the disadvantage of this treatment option is that there is only a 3–4.5 h treatment window (5). Presently, diagnosing IS mainly relies on typical clinical symptoms and brain imaging examinations (6). However, about 50% of IS lack specificity in early imaging diagnosis (7). In addition, the underlying mechanisms of stroke have not been entirely identified, and there are currently no blood biomarkers of IS for clinical use (8). Therefore, it is crucial to explore novel biomarkers of IS, which can contribute to a comprehensive understanding of the etiology and pathophysiology of the disease and aid in early diagnosis and treatment. Previously, miRNA and lncRNA have been proposed as possible stroke biomarkers (9, 10). In contrast, researchers have recently begun to investigate the role of circRNA in IS and search for IS-related circRNA biomarkers (11).

Circular RNA (circRNA) is a highly conserved and stable non-coding RNA with a circular structure that does not contain 5′ end caps and 3′ end poly (A) tails (12). CircRNAs have been reported to be extensively involved in the process of IS, including participating in ischemic brain injury, inhibiting apoptosis and neuroinflammation, and protecting the blood-brain barrier (13). In addition, exosomes can carry circRNA across the blood-brain barrier, allowing circRNA to exist stably in peripheral blood, making circRNA promising as a novel diagnostic and prognostic biomarker for IS (14). A recent study reported that CircOGDH expression was upregulated in the plasma of IS patients, and the CircOGDH expression was positively correlated with penumbra size (15). Li et al. examined circulating blood circRNA expression profiles of acute stroke patients and healthy controls and identified differentially expressed circRNAs in IS patients (16). These previous studies have indicated that peripheral circRNA expression levels are dysregulated in IS patients. Zuo et al. reported that down-regulation of circCDC14A in peripheral blood cells alleviates brain injury in the acute phase of stroke in tMCAO mice (17). Thus, modulating the level of dysregulated circRNAs might reverse the pathological process of IS. circRNA can perform the corresponding function as a competitive endogenous RNA (ceRNA), which means circRNA can bind competitively with miRNA like a sponge through base complementarity, thus affecting the target gene and regulating the level of encoded transcripts (18). A recent study reported that hsa_circ_0045932 (circUSP36) attenuates ischemic stroke injury through the miR-139-3p/SMAD3/Bcl2 signaling axis (19). Xu et al. (20) revealed that circSKA3 could compete for binding hsa-miR-6796-5p and thereby regulates MMP9 to promote ischemic stroke progression. However, there remain numerous dysregulated circRNAs whose roles in IS have not been reported. Thus, the potential role of circRNAs acting as ceRNAs involved in the process of IS is currently unclear.

Our study conducted a comprehensive analysis of the IS microarray data from the Gene Expression Omnibus (GEO) database. Eventually, we identified a novel potential circRNA-miRNA-mRNA regulatory network in IS. In addition, we screened out immune-related hub genes in the ceRNA network and further constructed an immune-related ceRNA sub-network. Our study provides a novel perspective on the molecular mechanisms of ceRNA immunoregulation in IS.

## Materials and Methods

### Microarray Data

Microarray datasets (GSE195442, GSE117064, and GSE58294) were downloaded from the Gene Expression Omnibus (GEO) database (https://www.ncbi.nlm.nih.gov/geo/) (21). The dataset GSE195442 is based on the GPL31275 Agilent-085499_SBC human ceRNA microarray [ProbeName version]. All samples in the GSE195442 were included in our study, containing plasma exosome samples from 10 IS patients and 10 controls. The dataset GSE117064 is based on the GPL21263 3D-Gene Human miRNA V21_1.0.0. It contains 1785 serum samples, out of which 189 male participants were collected from individuals aged >65 years, comprising 84 IS patients and 105 controls, and were included in our study. The dataset GSE58294 is based on the GPL570 [HG-U133_Plus_2] Affymetrix Human Genome U133 Plus 2.0 Array. Peripheral blood samples from 23 ischaemic stroke patients (5 h of onset) and 23 controls in GSE58294 were included in our study. In addition, GSE16561, based on GPL6883 Illumina HumanRef-8 v3.0 expression beadchip, containing peripheral blood samples from 39 IS patients and 24 controls, was applied for validation. The ethics committee approval or informed consent was not required in this study, as the data were publicly obtained from the GEO database.

### Differentially Expression Analysis

DEcircRNAs, DEmiRNAs, and DEmRNAs were screened by the “limma” package in R software (22). The threshold was set with *p* value <0.01 and |log_2_fold change| >1.2 to select DEcircRNAs from GSE195442. Next, the GSE117064 was analyzed with adjusted *p* value <0.05 and |log_2_fold change| >2 and set as the threshold for selecting DEmiRNAs. Finally, DEmRNAs were screened by the cut-off point of adjusted *p* value <0.05 and |log_2_fold change| >1 in GSE58294.

### Gene Ontology and KEGG Enrichment Analysis

The “clusterProfiler” package in R was applied for Gene Ontology (GO) and Kyoto Encyclopedia of Genes and Genomes (KEGG) pathway enrichment analysis (23). *p*-Values lower than 0.05 were considered statistically significant.

### PPI Network Construction and Hub Genes Identification

A PPI network of the DEmRNAs was established by the STRING database (http://string-db.org) (24). Interaction scores over 0.4 were considered statistically significant. Next, the interaction network was downloaded and imported into the Cytoscape software for visualization (25). Then, the top 20 genes ranked by Degree and MCC score were selected respectively by the cytoHubba plugin app (26).

### Construction of ceRNA Network in IS

We predicted the target miRNAs interacted with the DEcircRNAs by the Circular RNA Interactome (CircInteractome) database (https://circinteractome.nia.nih.gov/) (27). The predicted miRNAs were intersected with the DEmiRNAs by a Venn diagram *via* the online tool – Venny 2.1.0 (https://bioinfogp.cnb.csic.es/tools/venny/index.html). Then, the target mRNAs of the overlapping miRNAs were predicted by the miRDB database (28), and the intersection of the predicted mRNAs and DEmRNAs was obtained by Venny 2.1.0. Finally, the ceRNA network was constructed based on the expression of circRNAs, miRNAs, and mRNAs, and the results were visualized using Cytoscape software.

### Construction of Immune-Related ceRNA Sub-network

Immune-related genes (IRGs) were obtained from the ImmPort database (29). We intersected the IRGs, top 20 hub genes ranked by Degree, top 20 hub genes ranked by MCC, and target genes in the ceRNA network and identified immune-related hub genes. Then, we constructed an immune-related circRNA-miRNA-mRNA sub-network, which was visualized through the Cytoscape software.

### Diagnostic Analysis of mRNA of Immune-Related Sub-ceRNA Network

An independent external dataset, GSE16561, including 39 IS patients and 24 controls, was used for validation. The effectiveness of the target genes in the immune-related ceRNA sub-network was performed by the receiver operating characteristic (ROC) curves *via* the “pROC” package in R (30). In addition, the mRNA expression levels of immune-related hub genes were compared between IS patients and controls by t-test, and a *p* value < 0.05 was considered statistically significant.

### Estimation of the Subtype Distribution of Immune Cells

The relative expression of different immune cell subtypes in 46 samples (23 controls vs. 23 IS patients of 5 h onset) in GSE58294 was assessed with CIBERSORT in R (31). First, the proportion of each kind of immune cell in the 46 samples was shown by histogram. Then, the relative expression of every immune cell subtype was compared between the controls and IS group using a boxplot. Finally, the relationship between the immune-related hub genes and each immune cell was performed using Pearson correlation and visualized by a lollipop graph.

## Results

### Identification of DEcircRNAs, DEmiRNAs, and DEmRNAs in IS

**Figure 1:**
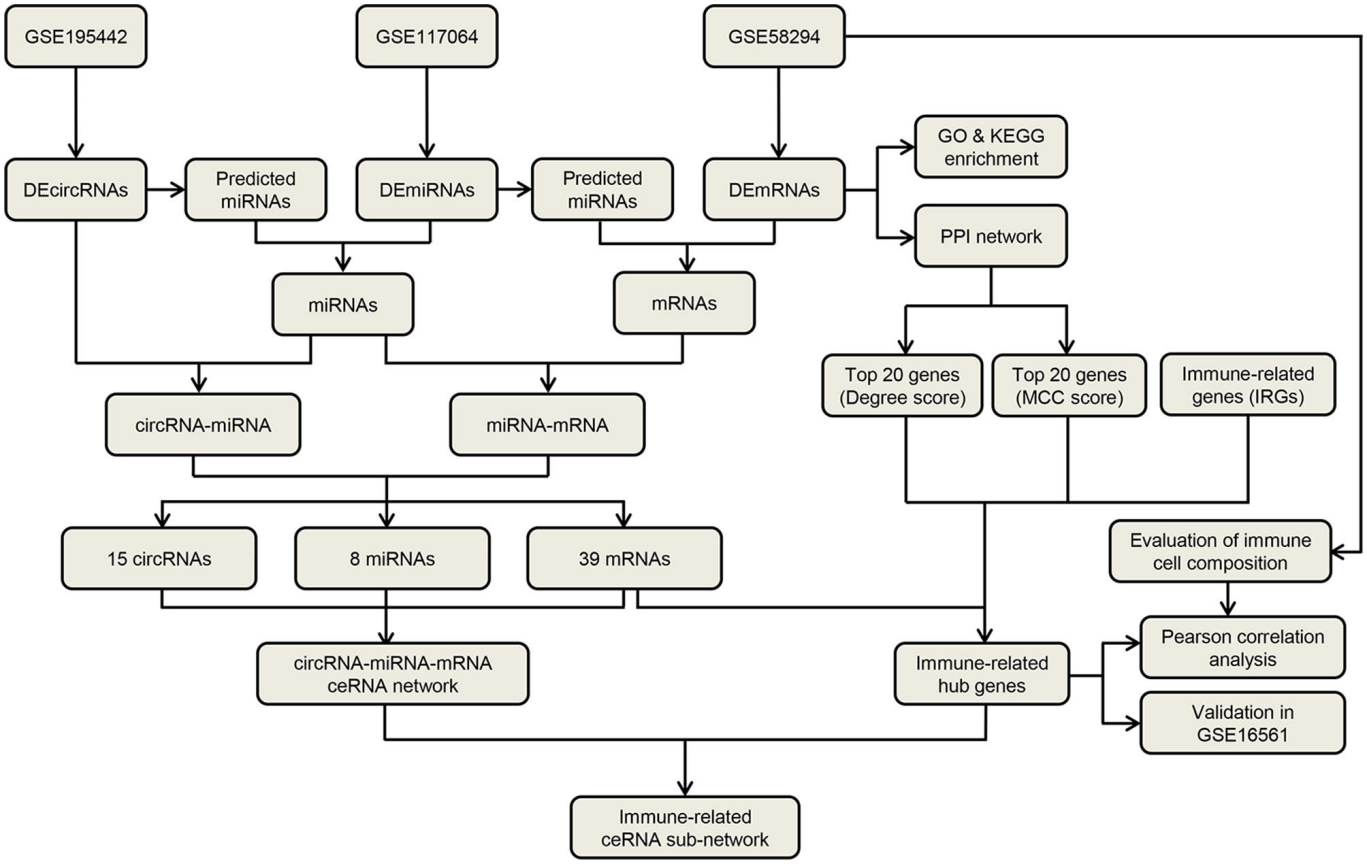
The flow diagram for the whole study.

**Figure 2:**
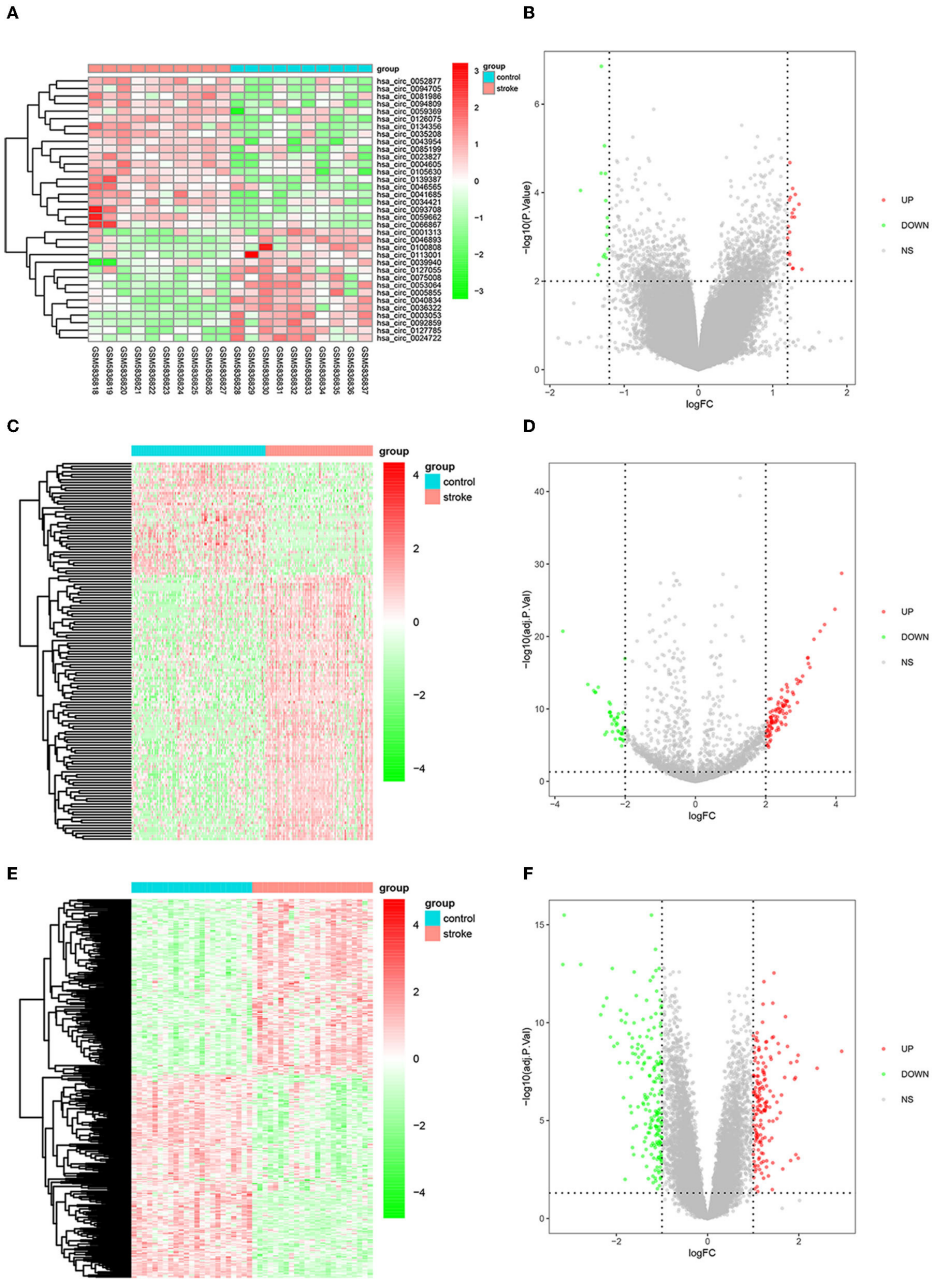
DEcircRNAs, DEmiRNAs, and DEmRNAs in IS. **(A)** Heatmap and **(B)** volcano map of DEcircRNAs in the GSE195442 dataset. **(C)** Heatmap and **(D)** volcano map of DEmiRNAs in the GSE117064 dataset. **(E)** Heatmap and **(F)** volcano map of DEmRNAs in the GSE58294 dataset.

The flow diagram for the whole study is shown in Figure 1. A total of 35 DEcircRNAs (20 up-regulated and 15 down-regulated) were obtained in the GSE195442 dataset (Figures 2A,B). In addition, 141 DEmiRNAs (99 up-regulated and 42 down-regulated) were identified in the GSE117064 dataset (Figures 2C,D). Furthermore, 356 DEmRNAs (165 up-regulated and 191 down-regulated) were screened in the GSE58294 dataset (Figures 2E,F). Lists of DEcircRNAs, DEmiRNAs, and DEmRNAs are available in Supplementary Tables S1–S3.

### Gene Ontology and KEGG Enrichment Analysis

**Figure 3:**
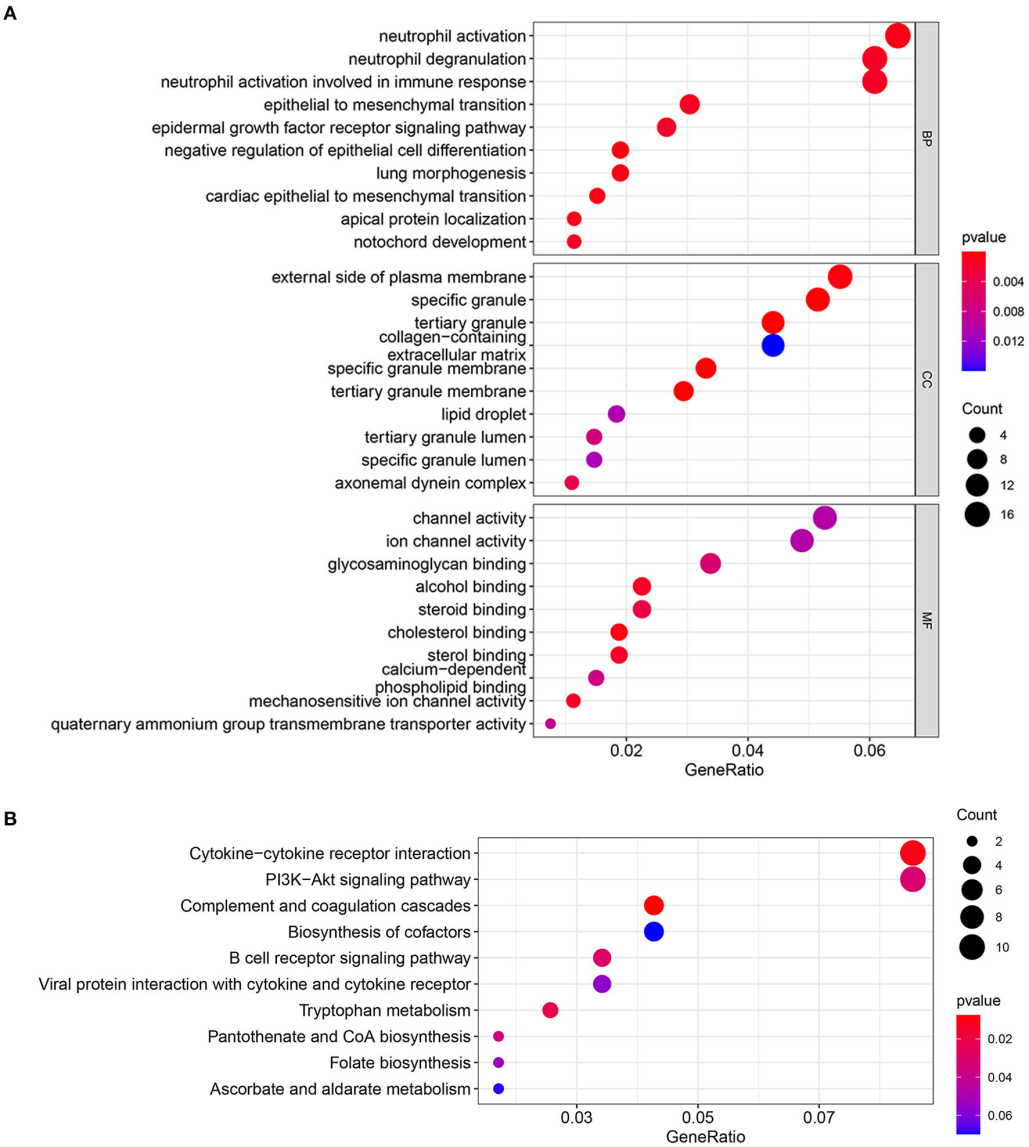
**(A)** GO enrichment analysis for DEmRNAs. **(B)** KEGG pathway enrichment analysis for DEmRNAs.

Gene Ontology terms are classified into three main categories: biological processes (BP), cellular components (CC), and molecular functions (MF). The top three terms in the BP contain neutrophil activation, neutrophil degranulation, and neutrophil activation involved in immune response. The top three terms in the CC contain external side of plasma membrane, specific granule, and tertiary granule. The top three terms in MF contains: channel activity, ion channel activity, and glycosaminoglycan binding (Figure 3A). The results of the KEGG enrichment pathway demonstrate that DEGs are principally involved in cytokine–cytokine receptor interaction, PI3K–Akt signaling pathway, and complement and coagulation cascades (Figure 3B).

### PPI Network Construction and Hub Genes Identification

**Figure 4:**
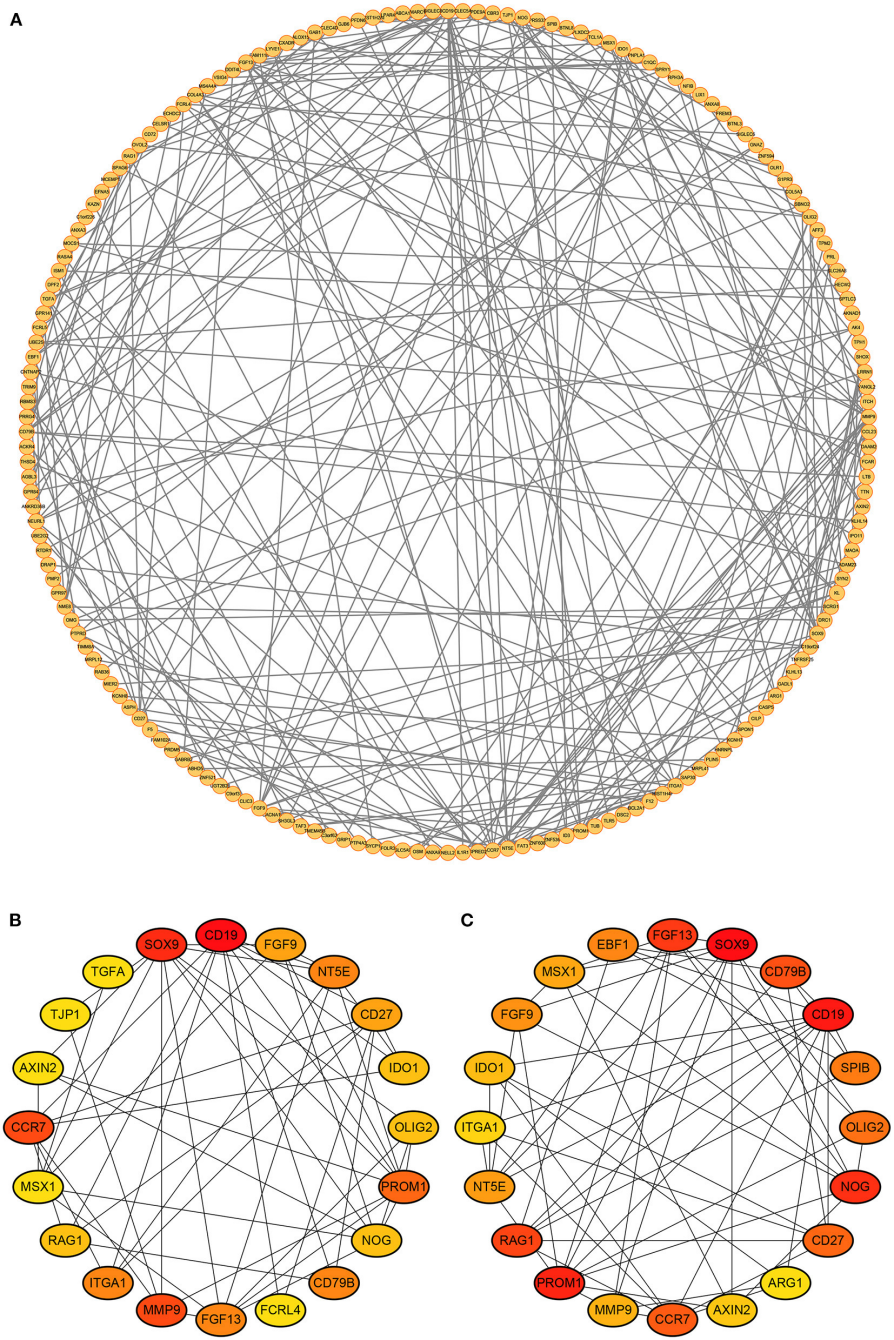
Protein-protein interactions network. **(A)** The PPI network of DEmRNAs. **(B)** Top 20 genes ranked by Degree score. **(C)** Top 20 genes ranked by MCC score.

The PPI network of the DEmRNAs was constructed by the STRING database and imported into the Cytoscape software (Figure 4A). According to the Degree score, the top 20 genes included CD19, CCR7, RAG1, OLIG2, ITGA1, FGF9, IDO1, AXIN2, SOX9, TGFA, CD27, NT5E, PROM1, FCRL4, TJP1, NOG, MMP9, MSX1, FGF13, and CD79B (Figure 4B). In addition, the top 20 genes ranked by MCC score contained CD19, CCR7, RAG1, OLIG2, ITGA1, ARG1, SPIB, FGF9, IDO1, AXIN2, SOX9, CD27, NT5E, PROM1, NOG, EBF1, MMP9, MSX1, FGF13, and CD79B (Figure 4C).

### Construction of the circRNA-miRNA-mRNA Regulatory Network

**Figure 5:**
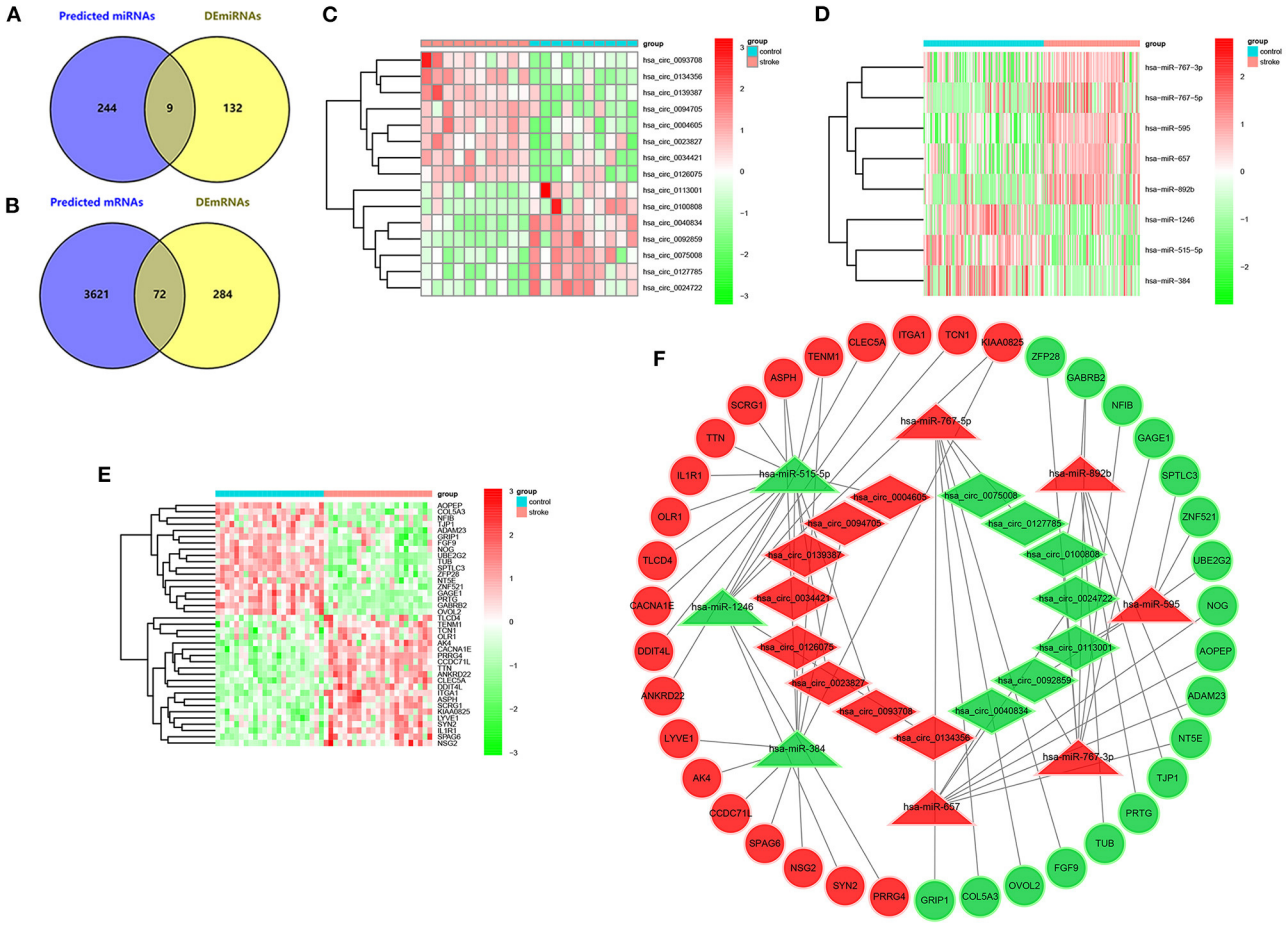
Constrcution of ceRNA network in IS. **(A)** Venn diagram of intersecting miRNAs of predicted miRNAs and DEmiRNAs in the GSE117064 dataset. **(B)** Venn diagram of intersecting mRNAs of predicted mRNAs and DEmRNAs in the GSE58294 dataset. **(C)** Heatmap of 15 DEcircRNAs involved in the ceRNA network in the GSE195442 dataset. **(D)** Heatmap of 8 DEmiRNAs involved in the ceRNA network in the GSE117064 dataset. **(E)** Heatmap of 39 DEmRNAs involved in the ceRNA network in the GSE58294 dataset. **(F)** circRNA-miRNA-mRNA network in IS. The diamond, triangle and circle indicate circRNA, miRNA and mRNA respectively. Red represents expression up-regulation and green represents expression down-regulation.

A total of 253 potential target miRNAs of the DEcircRNAs were predicted in the CircInteractome database. Then, nine overlapping miRNAs were obtained from the intersection of predicted miRNAs and DEmiRNAs (Figure 5A). Next, by using the miRDB database, 3,693 potential target genes for the overlapping miRNAs were identified. Subsequently, 72 intersecting mRNAs were acquired by the intersection of predicted target genes and DEmRNAs (Figure 5B). Finally, the ceRNA network of IS was constructed based on the negative regulation of the circRNA-miRNA pair and miRNA-mRNA pair, which included 15 circRNAs (Figure 5C), eight miRNAs (Figure 5D), and 39 mRNAs (Figure 5E). The ceRNA network is shown in Figure 5F.

### Immune-Related ceRNA Sub-network Construction

**Figure 6:**
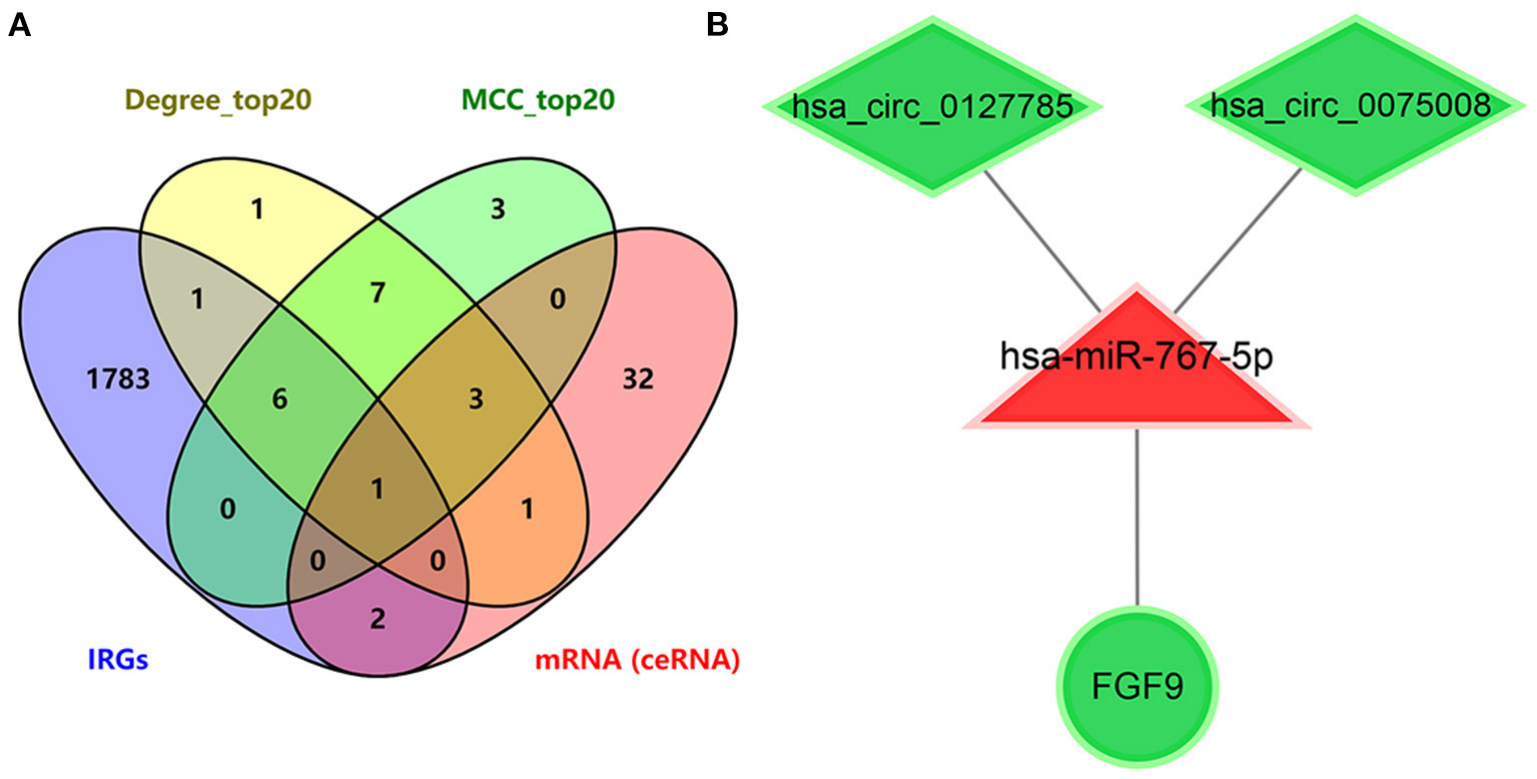
Identification of immume-related ceRNA subnetwork in IS. **(A)** Venn diagram of intersecting mRNA of IRGs, top 20 genes ranked by Degree score, top 20 genes ranked by MCC score, and mRNAs in the ceRNA network of IS. **(B)** Immume-related circRNA-miRNA-mRNA subnetwork.

**Figure 7:**
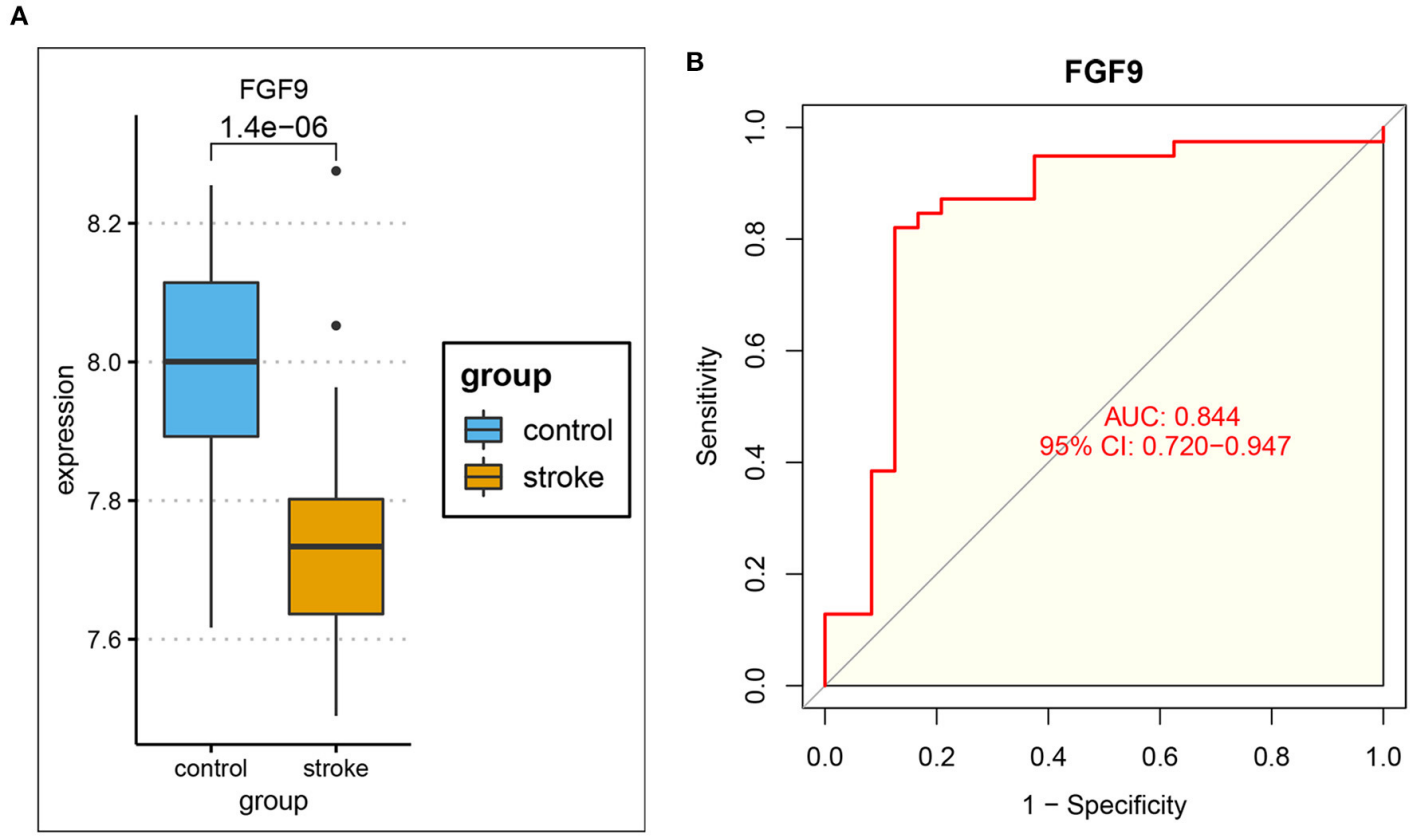
Validation of FGF9 in the independent dataset GSE16561. **(A)** The expression of FGF9 was significantly down-regulated in IS patients compared to controls. **(B)** ROC curve analysis indicated that the expression of FGF9 was significantly associated with the diagnosis of IS (AUC = 0.844).

To further explore immune-related ceRNA regulatory relationships in IS, we obtained immune-related genes (IRGs) in the ImmPort database and intersected the IRGs with mRNAs in the ceRNA network and the hub genes of DEmRNAs (Figure 6A). As a result, the overlapping gene FGF9 was identified as the immune-related hub gene, and the immune-related ceRNA subnetwork in IS was subsequently constructed. Finally, hsa_circ_0127785 and hsa_circ_0075008 (down-regulated in IS), hsa-miR-767-5p (up-regulated in IS), and FGF9 (down-regulated in IS) formed an immune-related ceRNA regulatory network (Figure 6B). In the independent dataset GSE16561, the expression of FGF9 in IS patients and controls was validated (Figure 7A), and FGF9 expression was significantly associated with a diagnosis of IS (AUC = 0.844) by ROC curve analysis (Figure 7B).

### Relative Immune Cells Expression

**Figure 8:**
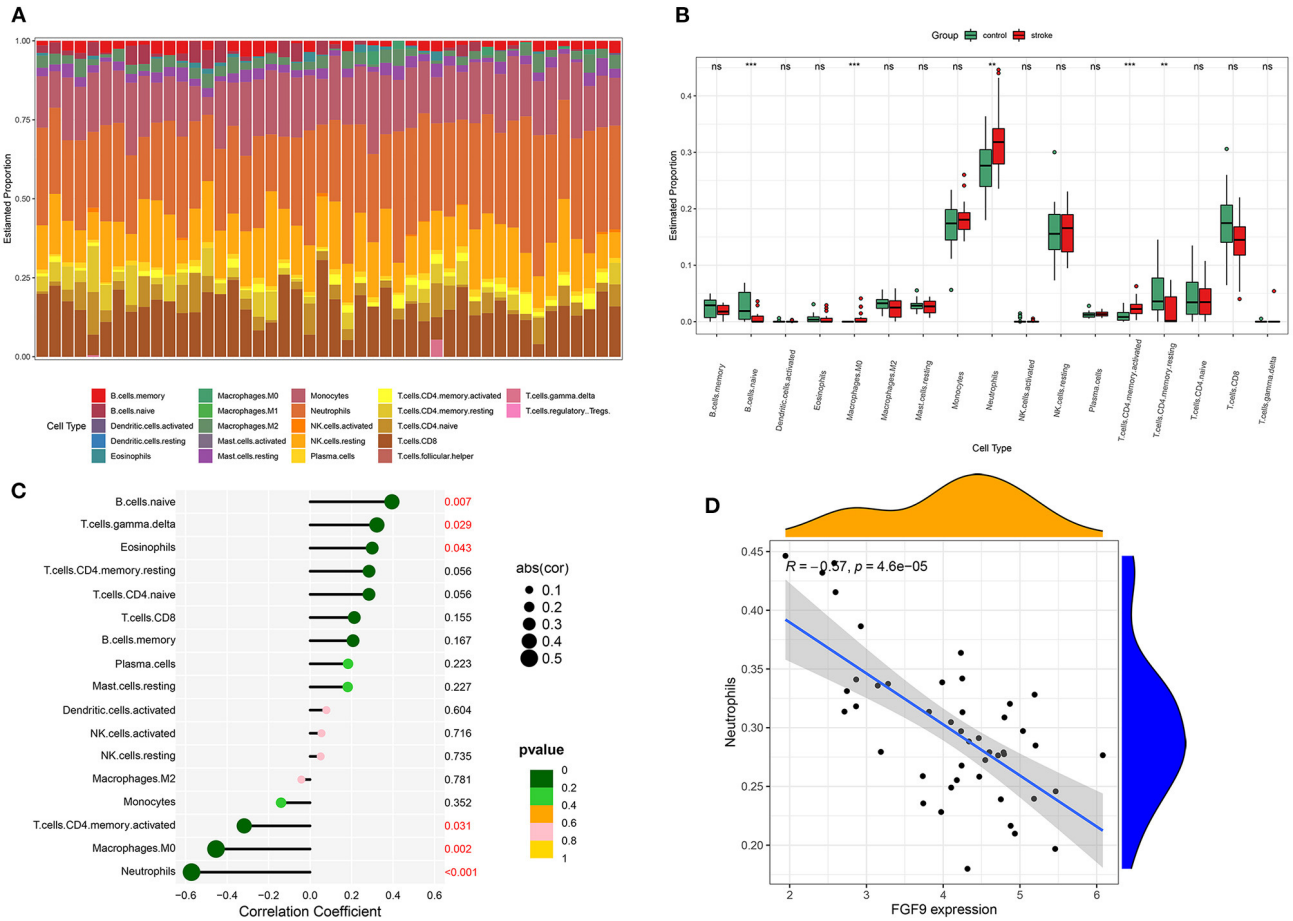
Relative expression of immune cell subtypes. **(A)** Histogram of the proportion of each kind of immune cell. **(B)** Boxplot of relative expression of every immune cell subtypes between the controls and IS group. **(C)** Lollipop graph of the correlation between the FGF9 expression and each immune cell subtype. **(D)** The level of neutrophils was significantly negatively correlated with FGF9 expression.

We analyzed the relative expression of 22 immune cells in each of the included samples of GSE58294 through CIBERSORT, and the result showed that 17 kinds of immune cells were expressed in the included samples. The distribution of immune cell subtypes in each sample is shown in Figure 8A. Neutrophils recorded the highest expression in all samples, and the expression was significantly higher in IS patients than in controls (Figure 8B). In addition, the expression of the immune-related hub gene, FGF9, was analyzed in correlation with the expression of each immune cell subtype. As a result, the expression of FGF9 was significantly negatively correlated with the level of neutrophils (*p* < 0.001; Figures 8C,D).

## Discussion

Stroke carries a severe burden on society and the economy (32). IS is the predominant type of stroke. Due to the narrow window for thrombolytic therapy and the frequent lack of specificity of imaging characteristics, it is essential to identify blood biomarkers for rapid diagnosis (33). In addition, identifying potentially relevant genes in stroke and screening for circRNA-miRNA-mRNA regulatory interactions contribute to further understanding of the pathogenesis of IS and provide new insights into the prevention, diagnosis, and treatment of IS.

Numerous studies revealed that abnormal expression of circRNAs is involved in various diseases (34). Recent studies have shown that circRNAs are involved in post-transcriptional regulation in IS (35). Yang et al. (36) revealed that circSCMH1 targeted delivered to the brain mediated by extracellular vesicle improved functional recovery after stroke in mice and monkeys. A clinical trial discovered significant upregulation of circPDS5B and circCDC14A in IS patients (37). Therefore, circRNA is a promising novel target for the diagnosis and prognosis of IS. CircRNA can sponge miRNA, thus reducing miRNA regulation on downstream target genes (38). However, the mechanism of ceRNA in IS is not fully understood, and an increasing number of studies are focusing on the role of circRNA-miRNA-mRNA in IS (39). There is plenty of space to extend further studies on the role of circRNA as ceRNA in IS.

In this study, we identified 35 DEcircRNAs, 141 DEmiRNAs, and 356 DEmRNAs in the expression profiles from the GEO database. Then, we screened circRNA-miRNA interaction pairs through the CircIteractome database and miRNA-mRNA interaction pairs through the miRDB database. By intersecting the predicted miRNAs and DEmiRNAs, and intersecting the predicted mRNAs and DEmRNAs, were finally identified a potential circRNA-miRNA-mRNA regulatory network in IS, which contained 15circRNAs (eight up-regulated and seven down-regulated), eight miRNAs (five up-regulated and three down-regulated), and 39 mRNAs (21 up-regulated and 18 down-regulated).

To understand the function of DEmRNAs in IS, we performed the Gene Ontology and KEGG pathway enrichment analysis. As a result, neutrophil activation was significantly enriched in the BP module of GO, and cytokine–cytokine receptor interaction was significantly enriched by KEGG pathway enrichment. Enrichment results confirm the reliability of DEmRNAs, as numerous studies have suggested that the inflammatory response has an essential role in the various phases after the onset of IS, which affects the prognosis and treatment of stroke (40). Thus we further downloaded the immune-related genes (IRGs) from the ImmPort database to screen for the immune-related hub genes in IS. As a result, FGF9, as an intersection of IRGs, PPI hub genes, and target genes in the ceRNA network, was identified as a target gene for constructing immune-related ceRNA sub-network. The sub-network contains hsa_circ_0127785/hsa_circ_0075008 (down-regulated expression in IS), hsa-miR-767-5p (up-regulated expression in IS), and FGF9 (down-regulated expression in IS). The expression pattern of the target gene FGF9 was validated in the independent dataset GSE16561. As a result, FGF9 remained significantly down-regulated expression in IS patients, and the ROC curve indicated a good diagnostic efficacy (AUC = 0.844).

The CIBERSORT algorithm revealed a significant increase in the level of neutrophils after the onset of IS, consistent with previous studies. Studies indicated that properties of circulating neutrophils significantly increased after IS, and the neutrophil alterations were notably correlated with the IS severity (41). An elevated neutrophil to lymphocyte ratio may signal a poor prognosis for IS (42). However, As one of the earliest immune cells to be recruited into the ischaemic brain, neutrophils are involved in a double-edged role in the pathology of IS (43). In addition, the expression of FGF9 was significantly and negatively correlated with the level of neutrophils by Pearson correlation coefficients, which suggested an essential effect of FGF9 in ischemic stroke.

Interestingly, certain recent studies indicated the potential role of FGF9 in IS. It has been reported that long non-coding RNA SNHG7 can mediate up-regulation of FGF9 to alleviate the oxygen and glucose deprivation-induced neuron cell injury (44). Downregulation of FGF9 mediated by miR-339 promotes hypoxia-induced neuronal apoptosis and impairs cell viability (45). These results suggest that FGF9 appears to be a promising target for the diagnosis and treatment of IS. Multiple studies have reported the involvement of hsa-miR-767-5p in cancer. Feng et al. reported that over-expression of hsa-miR-767-5p promotes tumor progression in multiple myeloma model mice, and this effect can be suppressed by CircRNA circ_0000190 (46). Meng et al. suggested that LINC-PINT downregulates miR-767-5p to induce TET2 expression, thus suppressing the aggressiveness of thyroid cancer. However, the role of miR - 767-5p in IS has been rarely reported. Herein, we identified a novel immune-related circRNA-miRNA-mRNA regulatory network. We hypothesized that circ_0127785/ circ_0075008/miR-767-5p/FGF9 is involved in the process of ischemic stroke and brings novel insight into the diagnosis and treatment of IS. However, the findings were obtained based on bioinformatic analysis. The regulatory relationships in the ceRNA network remain experimental confirmation.

## Conclusion

We identified a potential circRNA-miRNA-mRNA regulatory network in IS, including 15 DEcircRNAs, eight DEmiRNAs, and 39 DEmRNAs. In addition, circ_0127785/ circ_0075008/miR-767-5p/FGF9 was identified as an immune-related regulatory sub-network of IS. Our study provides new insights into the pathological processes of IS mediated by circRNA, and the current findings require validation in the future.

## Data Availability Statement

The original contributions presented in the study are included in the article/Supplementary Material, further inquiries can be directed to the corresponding author.

## Author Contributions

SC designed the study and wrote the manuscript. YZ assisted in analyzing the data and revising the manuscript. MC and WO critically read and edited the manuscript. All authors contributed to the article and approved the submitted version.

## Funding

This study was supported by the National Natural Science Foundation of China (No. 81971028).

## Conflict of Interest

The authors declare that the research was conducted in the absence of any commercial or financial relationships that could be construed as a potential conflict of interest.

## Publisher's Note

All claims expressed in this article are solely those of the authors and do not necessarily represent those of their affiliated organizations, or those of the publisher, the editors and the reviewers. Any product that may be evaluated in this article, or claim that may be made by its manufacturer, is not guaranteed or endorsed by the publisher.

## References

[1] Campbell BCV De Silva DA Macleod MR Coutts SB Schwamm LH Davis SM. Ischaemic stroke. Nat Rev Dis Primers. (2019) 5:70. 10.1038/s41572-019-0118-8 31601801

[2] Collaborators GBDN. Global, regional, and national burden of neurological disorders, 1990-2016: a systematic analysis for the Global Burden of Disease Study 2016. Lancet Neurol. (2019) 18:459–80. 10.1016/S1474-4422(18)30499-X 30879893 PMC6459001

[3] Favate AS Younger DS. Epidemiology of ischemic stroke. Neurol Clin. (2016) 34:967–80. 10.1016/j.ncl.2016.06.013 27720004

[4] Benjamin EJ Virani SS Callaway CW Chamberlain AM Chang AR Cheng S. Heart disease and stroke statistics-2018 update: a report from the American Heart Association. Circulation. (2018) 137:e67–492. 10.1161/CIR.0000000000000573 29386200

[5] Cai Y Zhang Y Ke X Guo Y Yao C Tang N. Transcriptome sequencing unravels potential biomarkers at different stages of cerebral ischemic stroke. Front Genet. (2019) 10:814. 10.3389/fgene.2019.00814 31681398 PMC6798056

[6] Campbell BCV Khatri P. Stroke. Lancet. (2020) 396:129–42. 10.1016/S0140-6736(20)31179-X 32653056

[7] Hopyan J Ciarallo A Dowlatshahi D Howard P John V Yeung R. Certainty of stroke diagnosis: incremental benefit with Ct perfusion over noncontrast Ct and Ct angiography. Radiology. (2010) 255:142–53. 10.1148/radiol.09091021 20308452

[8] Whiteley W Tseng MC Sandercock P. Blood biomarkers in the diagnosis of ischemic stroke: a systematic review. Stroke. (2008) 39:2902–9. 10.1161/STROKEAHA.107.511261 18658039

[9] Mirzaei H Momeni F Saadatpour L Sahebkar A Goodarzi M Masoudifar A. MicroRNA: relevance to stroke diagnosis, prognosis, and therapy. J Cell Physiol. (2018) 233:856–65. 10.1002/jcp.25787 28067403

[10] Wang Q Liu X Zhu R. Long noncoding RNAs as diagnostic and therapeutic targets for ischemic stroke. Curr Pharm Des. (2019) 25:1115–21. 10.2174/1381612825666190328112844 30919772

[11] Liu M Liu X Zhou M Guo S Sun K. Impact of circRNAs on ischemic stroke. Aging Dis. (2022) 13:329–39. 10.14336/AD.2021.1113 35371609 PMC8947829

[12] Salzman J. Circular RNA expression: its potential regulation and function. Trends Genet. (2016) 32:309–16. 10.1016/j.tig.2016.03.002 27050930 PMC4948998

[13] Wang Q Liu X Zhao J Zhu R. Circular RNAs: novel diagnostic and therapeutic targets for ischemic stroke. Expert Rev Mol Diagn. (2020) 20:1039–49. 10.1080/14737159.2020.1826313 32954841

[14] Li JY Li QQ Sheng R. The role and therapeutic potential of exosomes in ischemic stroke. Neurochem Int. (2021) 151:105194. 10.1016/j.neuint.2021.105194 34582960

[15] Liu Y Li Y Zang J Zhang T Li Y Tan Z. CircOGDH is a penumbra biomarker and therapeutic target in acute ischemic stroke. Circ Res. (2022) 130:907–24. 10.1161/CIRCRESAHA.121.319412 35189704

[16] Li S Chen L Xu C Qu X Qin Z Gao J. Expression profile and bioinformatics analysis of circular RNAs in acute ischemic stroke in a South Chinese Han population. Sci Rep. (2020) 10:10138. 10.1038/s41598-020-66990-y 32576868 PMC7311391

[17] Zuo L Xie J Liu Y Leng S Zhang Z Yan F. Down-regulation of circular RNA Cdc14a peripherally ameliorates brain injury in acute phase of ischemic stroke. J Neuroinflammation. (2021) 18:283. 10.1186/s12974-021-02333-6 34876161 PMC8653620

[18] Qu X Li Z Chen J Hou L. The emerging roles of circular RNAs in CNS injuries. J Neurosci Res. (2020) 98:1485–97. 10.1002/jnr.24591 32052488

[19] Yang J He W Gu L Long J Zhu L Zhang R. Circusp36 attenuates ischemic stroke injury through the Mir-139-3p/Smad3/Bcl2 signal axis. Clin Sci. (2022). 10.1042/CS20220157. [Epub ahead of print]. 35532376

[20] Xu T Li Y Zhu N Su Y Li J Ke K. Circska3 acts as a sponge of Mir-6796-5p to be associated with outcomes of ischemic stroke by regulating matrix metalloproteinase 9 expression. Eur J Neurol. (2022) 29:486–95. 10.1111/ene.15164 34725884

[21] Edgar R Domrachev M Lash AE. Gene expression omnibus: NCBI gene expression and hybridization array data repository. Nucleic Acids Res. (2002) 30:207–10. 10.1093/nar/30.1.207 11752295 PMC99122

[22] Ritchie ME Phipson B Wu D Hu Y Law CW Shi W. Limma powers differential expression analyses for RNA-sequencing and microarray studies. Nucleic Acids Res. (2015) 43:e47. 10.1093/nar/gkv007 25605792 PMC4402510

[23] Yu G Wang LG Han Y He QY. Clusterprofiler: an R package for comparing biological themes among gene clusters. OMICS. (2012) 16:284–7. 10.1089/omi.2011.0118 22455463 PMC3339379

[24] Szklarczyk D Morris JH Cook H Kuhn M Wyder S Simonovic M. The string database in 2017: quality-controlled protein-protein association networks, made broadly accessible. Nucleic Acids Res. (2017) 45:D362–8. 10.1093/nar/gkw937 27924014 PMC5210637

[25] Shannon P Markiel A Ozier O Baliga NS Wang JT Ramage D. Cytoscape: a software environment for integrated models of biomolecular interaction networks. Genome Res. (2003) 13:2498–504. 10.1101/gr.1239303 14597658 PMC403769

[26] Chin CH Chen SH Wu HH Ho CW Ko MT Lin CY. Cytohubba: identifying hub objects and sub-networks from complex interactome. BMC Syst Biol. (2014) 8(Suppl 4):S11. 10.1186/1752-0509-8-S4-S11 25521941 PMC4290687

[27] Dudekula DB Panda AC Grammatikakis I De S Abdelmohsen K Gorospe M. Circinteractome: a web tool for exploring circular RNAs and their interacting proteins and microRNAs. RNA Biol. (2016) 13:34–42. 10.1080/15476286.2015.1128065 26669964 PMC4829301

[28] Chen Y Wang X. Mirdb: an online database for prediction of functional microRNA targets. Nucleic Acids Res. (2020) 48:D127–31. 10.1093/nar/gkz757 31504780 PMC6943051

[29] Bhattacharya S Andorf S Gomes L Dunn P Schaefer H Pontius J. Immport: disseminating data to the public for the future of immunology. Immunol Res. (2014) 58:234–9. 10.1007/s12026-014-8516-1 24791905

[30] Robin X Turck N Hainard A Tiberti N Lisacek F Sanchez JC. Proc: an open-source package for R and S+ to analyze and compare roc curves. BMC Bioinformatics. (2011) 12:77. 10.1186/1471-2105-12-77 21414208 PMC3068975

[31] Newman AM Liu CL Green MR Gentles AJ Feng W Xu Y. Robust enumeration of cell subsets from tissue expression profiles. Nat Methods. (2015) 12:453–7. 10.1038/nmeth.3337 25822800 PMC4739640

[32] Feigin VL Norrving B Mensah GA. Global burden of stroke. Circ Res. (2017) 120:439–48. 10.1161/CIRCRESAHA.116.308413 28154096

[33] Musuka TD Wilton SB Traboulsi M Hill MD. Diagnosis and management of acute ischemic stroke: speed is critical. CMAJ. (2015) 187:887–93. 10.1503/cmaj.140355 26243819 PMC4562827

[34] Kristensen LS Andersen MS Stagsted LVW Ebbesen KK Hansen TB Kjems J. The biogenesis, biology and characterization of circular RNAs. Nat Rev Genet. (2019) 20:675–91. 10.1038/s41576-019-0158-7 31395983

[35] Zhang X Hamblin MH Yin KJ. Noncoding RNAs and stroke. Neuroscientist. (2019) 25:22–6. 10.1177/1073858418769556 29637805 PMC7304476

[36] Yang L Han B Zhang Z Wang S Bai Y Zhang Y. Extracellular vesicle-mediated delivery of circular RNA Scmh1 promotes functional recovery in rodent and nonhuman primate ischemic stroke models. Circulation. (2020) 142:556–74. 10.1161/CIRCULATIONAHA.120.045765 32441115

[37] Zuo L Zhang L Zu J Wang Z Han B Chen B. Circulating circular RNAs as biomarkers for the diagnosis and prediction of outcomes in acute ischemic stroke. Stroke. (2020) 51:319–23. 10.1161/STROKEAHA.119.027348 31690252

[38] Sen R Ghosal S Das S Balti S Chakrabarti J. Competing endogenous RNA: the key to posttranscriptional regulation. Sci World J. (2014) 2014:896206. 10.1155/2014/896206 24672386 PMC3929601

[39] Lu S Yang X Wang C Chen S Lu S Yan W. Current status and potential role of circular RNAs in neurological disorders. J Neurochem. (2019) 150:237–48. 10.1111/jnc.14724 31099046

[40] Anrather J Iadecola C. Inflammation and stroke: an overview. Neurotherapeutics. (2016) 13:661–70. 10.1007/s13311-016-0483-x 27730544 PMC5081118

[41] Weisenburger-Lile D Dong Y Yger M Weisenburger G Polara GF Chaigneau T. Harmful neutrophil subsets in patients with ischemic stroke: association with disease severity. Neurol Neuroimmunol Neuroinflamm. (2019) 6:e571. 10.1212/NXI.0000000000000571 31355307 PMC6624098

[42] Wang L Song Q Wang C Wu S Deng L Li Y. Neutrophil to lymphocyte ratio predicts poor outcomes after acute ischemic stroke: a cohort study and systematic review. J Neurol Sci. (2019) 406:116445. 10.1016/j.jns.2019.116445 31521961

[43] Iadecola C Buckwalter MS Anrather J. Immune responses to stroke: mechanisms, modulation, and therapeutic potential. J Clin Invest. (2020) 130:2777–88. 10.1172/JCI135530 32391806 PMC7260029

[44] Sun W Sun L Sun X Ma S. Long non-coding RNA Snhg7 upregulates Fgf9 to alleviate oxygen and glucose deprivation-induced neuron cell injury in a Mir-134-5p-dependent manner. Metab Brain Dis. (2021) 36:2483–94. 10.1007/s11011-021-00852-y 34661812

[45] Gao XZ Ma RH Zhang ZX. Mir-339 promotes hypoxia-induced neuronal apoptosis and impairs cell viability by targeting Fgf9/Cacng2 and mediating Mapk pathway in ischemic stroke. Front Neurol. (2020) 11:436. 10.3389/fneur.2020.00436 32587563 PMC7297914

[46] Feng Y Zhang L Wu J Khadka B Fang Z Gu J. CircRNA Circ_0000190 inhibits the progression of multiple myeloma through modulating Mir-767-5p/Mapk4 pathway. J Exp Clin Cancer Res. (2019) 38:54. 10.1186/s13046-019-1071-9 30728056 PMC6364482

