# Supplementary material for: Integrated Analysis of Immune-Related circRNA-miRNA-mRNA Regulatory Network in Ischemic Stroke
Source: Front Neurol. 2022 Jun 15;13:889855. doi: 10.3389/fneur.2022.889855 (PMC9240315; doi:10.3389/fneur.2022.889855)
Supplement: Supplementary file 1 [file Table_1.DOCX]

**Table S1. List of 35 DEcircRNAs in the GSE195442 dataset**

| **DEcircRNAs** | **log_2_Fold Change** | ***p v*alue** |
| --- | --- | --- |
| hsa_circ_0036322 | -1.58744 | 8.92E-05 |
| hsa_circ_0039940 | -1.35394 | 0.007191 |
| hsa_circ_0113001 | -1.34067 | 0.004272 |
| hsa_circ_0092859 | -1.30983 | 3.64E-05 |
| hsa_circ_0003053 | -1.30668 | 1.39E-07 |
| hsa_circ_0100808 | -1.2796 | 0.002776 |
| hsa_circ_0040834 | -1.26466 | 8.74E-06 |
| hsa_circ_0005855 | -1.26237 | 0.002488 |
| hsa_circ_0127785 | -1.25219 | 3.69E-05 |
| hsa_circ_0053064 | -1.24738 | 0.000151 |
| hsa_circ_0001313 | -1.24415 | 0.002959 |
| hsa_circ_0024722 | -1.23699 | 0.000904 |
| hsa_circ_0046893 | -1.22618 | 0.000369 |
| hsa_circ_0075008 | -1.22425 | 0.000598 |
| hsa_circ_0127055 | -1.21691 | 0.001884 |
| hsa_circ_0105630 | 1.200363 | 0.001059 |
| hsa_circ_0023827 | 1.205389 | 0.002425 |
| hsa_circ_0034421 | 1.208811 | 0.000582 |
| hsa_circ_0081986 | 1.222886 | 0.000148 |
| hsa_circ_0126075 | 1.228321 | 0.000781 |
| hsa_circ_0046565 | 1.229444 | 0.004168 |
| hsa_circ_0085199 | 1.229857 | 0.002215 |
| hsa_circ_0093708 | 1.23198 | 0.002523 |
| hsa_circ_0134356 | 1.231986 | 2.10E-05 |
| hsa_circ_0035208 | 1.238012 | 0.000128 |
| hsa_circ_0094809 | 1.259585 | 0.000356 |
| hsa_circ_0094705 | 1.263313 | 0.00029 |
| hsa_circ_0059369 | 1.268059 | 0.005168 |
| hsa_circ_0004605 | 1.269171 | 8.06E-05 |
| hsa_circ_0043954 | 1.275586 | 0.005097 |
| hsa_circ_0059662 | 1.285946 | 0.000241 |
| hsa_circ_0139387 | 1.292597 | 0.000357 |
| hsa_circ_0052877 | 1.304696 | 0.00011 |
| hsa_circ_0041685 | 1.354102 | 0.000183 |
| hsa_circ_0066867 | 1.392738 | 0.005406 |

**Table S2. List of 141 DEmiRNAs in the GSE117064 dataset**

| **DEmiRNAs** | **log_2_Fold Change** | **adj. *p* value** |
| --- | --- | --- |
| hsa-miR-4485-3p | -3.77376 | 1.91E-21 |
| hsa-miR-17-5p | -3.06196 | 4.17E-14 |
| hsa-miR-137 | -2.89443 | 3.03E-13 |
| hsa-miR-221-3p | -2.85631 | 5.18E-13 |
| hsa-miR-6869-3p | -2.83858 | 5.10E-13 |
| hsa-miR-5693 | -2.76522 | 1.01E-13 |
| hsa-miR-1246 | -2.57565 | 3.48E-07 |
| hsa-let-7g-5p | -2.52884 | 1.58E-07 |
| hsa-miR-548az-3p | -2.45519 | 1.19E-11 |
| hsa-miR-516a-5p | -2.44568 | 2.99E-10 |
| hsa-miR-323a-3p | -2.43331 | 2.69E-10 |
| hsa-miR-127-3p | -2.42654 | 3.02E-10 |
| hsa-miR-22-3p | -2.42224 | 2.15E-11 |
| hsa-miR-4757-3p | -2.40348 | 1.12E-09 |
| hsa-miR-4519 | -2.40264 | 8.47E-10 |
| hsa-miR-106a-3p | -2.37101 | 1.22E-08 |
| hsa-miR-16-5p | -2.36045 | 1.43E-07 |
| hsa-miR-3687 | -2.31408 | 3.96E-09 |
| hsa-miR-523-3p | -2.3125 | 2.25E-09 |
| hsa-let-7a-5p | -2.27784 | 1.52E-06 |
| hsa-miR-518f-5p | -2.26012 | 8.29E-09 |
| hsa-miR-362-5p | -2.25759 | 1.83E-08 |
| hsa-miR-597-5p | -2.22157 | 1.22E-08 |
| hsa-miR-371a-3p | -2.21992 | 1.32E-09 |
| hsa-miR-6837-3p | -2.2199 | 1.66E-09 |
| hsa-miR-195-5p | -2.20701 | 1.08E-07 |
| hsa-miR-6838-5p | -2.19012 | 4.81E-10 |
| hsa-miR-181a-5p | -2.17439 | 1.43E-07 |
| hsa-miR-24-1-5p | -2.15364 | 1.28E-06 |
| hsa-miR-191-5p | -2.12789 | 1.45E-06 |
| hsa-miR-1973 | -2.11122 | 2.69E-07 |
| hsa-miR-409-5p | -2.10522 | 2.52E-07 |
| hsa-let-7d-5p | -2.09886 | 1.28E-05 |
| hsa-miR-21-5p | -2.07244 | 1.69E-06 |
| hsa-miR-384 | -2.06981 | 9.02E-08 |
| hsa-miR-515-5p | -2.06707 | 2.71E-07 |
| hsa-miR-3622b-3p | -2.06342 | 3.20E-10 |
| hsa-miR-376c-3p | -2.05678 | 3.99E-08 |
| hsa-miR-19b-3p | -2.05258 | 2.59E-06 |
| hsa-miR-186-3p | -2.03141 | 6.30E-08 |
| hsa-miR-4668-3p | -2.02045 | 4.17E-08 |
| hsa-miR-4800-3p | -2.00706 | 1.16E-17 |
| hsa-miR-6499-3p | 2.002157 | 5.21E-07 |
| hsa-miR-6872-5p | 2.009789 | 1.64E-07 |
| hsa-miR-5093 | 2.018089 | 5.56E-06 |
| hsa-miR-4474-3p | 2.027248 | 5.93E-07 |
| hsa-miR-4670-5p | 2.030637 | 1.08E-05 |
| hsa-miR-6512-3p | 2.033029 | 9.77E-08 |
| hsa-miR-1266-5p | 2.038436 | 5.38E-09 |
| hsa-miR-4784 | 2.03909 | 8.97E-08 |
| hsa-miR-550a-3p | 2.059356 | 3.30E-08 |
| hsa-miR-5006-3p | 2.060491 | 1.19E-08 |
| hsa-miR-6130 | 2.067785 | 1.02E-05 |
| hsa-miR-6822-3p | 2.076239 | 6.72E-07 |
| hsa-miR-4709-5p | 2.083145 | 1.90E-05 |
| hsa-miR-3692-5p | 2.089846 | 4.37E-12 |
| hsa-miR-758-5p | 2.090434 | 8.47E-08 |
| hsa-miR-3935 | 2.092802 | 4.41E-07 |
| hsa-miR-5681b | 2.09684 | 2.39E-07 |
| hsa-miR-6823-5p | 2.111128 | 3.41E-06 |
| hsa-miR-584-3p | 2.113244 | 2.36E-08 |
| hsa-miR-4711-3p | 2.126095 | 1.49E-07 |
| hsa-miR-5580-5p | 2.130082 | 2.83E-10 |
| hsa-miR-1306-3p | 2.131735 | 1.34E-09 |
| hsa-miR-4639-3p | 2.133784 | 2.05E-08 |
| hsa-miR-670-5p | 2.137239 | 1.99E-06 |
| hsa-miR-4518 | 2.142424 | 1.63E-09 |
| hsa-miR-1298-3p | 2.143125 | 4.06E-07 |
| hsa-miR-187-3p | 2.148233 | 3.85E-12 |
| hsa-miR-4701-3p | 2.151825 | 5.93E-09 |
| hsa-miR-449c-3p | 2.155126 | 3.45E-09 |
| hsa-miR-6884-5p | 2.156579 | 1.51E-09 |
| hsa-miR-4642 | 2.156755 | 7.39E-09 |
| hsa-miR-767-3p | 2.161369 | 8.09E-09 |
| hsa-miR-4694-3p | 2.170464 | 5.14E-08 |
| hsa-miR-3138 | 2.175916 | 4.44E-08 |
| hsa-miR-551b-3p | 2.175936 | 8.58E-09 |
| hsa-miR-212-5p | 2.177927 | 3.59E-09 |
| hsa-miR-3691-5p | 2.192223 | 5.28E-09 |
| hsa-miR-3189-3p | 2.194206 | 8.43E-10 |
| hsa-miR-6859-5p | 2.197088 | 1.79E-10 |
| hsa-miR-3927-5p | 2.215457 | 8.55E-09 |
| hsa-miR-3591-3p | 2.26839 | 1.02E-09 |
| hsa-miR-6833-5p | 2.276832 | 7.54E-09 |
| hsa-miR-5004-5p | 2.277891 | 5.27E-09 |
| hsa-miR-550b-3p | 2.285865 | 1.24E-10 |
| hsa-miR-6867-5p | 2.293588 | 2.78E-09 |
| hsa-miR-6878-3p | 2.293822 | 1.98E-10 |
| hsa-miR-6083 | 2.304754 | 9.50E-08 |
| hsa-miR-4669 | 2.332624 | 1.29E-09 |
| hsa-miR-5584-3p | 2.332818 | 4.06E-09 |
| hsa-miR-3617-3p | 2.338361 | 1.02E-10 |
| hsa-miR-6764-5p | 2.347676 | 2.29E-11 |
| hsa-miR-4801 | 2.351508 | 3.88E-08 |
| hsa-miR-6873-3p | 2.361823 | 1.13E-11 |
| hsa-miR-1178-3p | 2.367198 | 7.41E-08 |
| hsa-miR-608 | 2.375995 | 7.87E-11 |
| hsa-miR-3198 | 2.384464 | 2.83E-10 |
| hsa-miR-1910-3p | 2.409376 | 2.75E-10 |
| hsa-miR-466 | 2.417535 | 1.15E-10 |
| hsa-miR-3150b-3p | 2.433268 | 6.85E-09 |
| hsa-miR-4692 | 2.446991 | 2.61E-12 |
| hsa-miR-5685 | 2.456662 | 2.28E-13 |
| hsa-miR-4296 | 2.459279 | 1.77E-11 |
| hsa-miR-7156-5p | 2.459299 | 1.27E-08 |
| hsa-miR-5708 | 2.47193 | 1.67E-10 |
| hsa-miR-6504-5p | 2.48448 | 4.50E-11 |
| hsa-miR-6855-3p | 2.491331 | 3.24E-11 |
| hsa-miR-4712-5p | 2.504804 | 4.33E-10 |
| hsa-miR-130b-5p | 2.506161 | 4.50E-11 |
| hsa-miR-657 | 2.536292 | 6.39E-12 |
| hsa-miR-4502 | 2.549092 | 1.46E-09 |
| hsa-miR-4420 | 2.550565 | 2.22E-11 |
| hsa-miR-4456 | 2.567849 | 3.24E-11 |
| hsa-miR-767-5p | 2.568744 | 1.52E-08 |
| hsa-miR-3667-3p | 2.578118 | 9.46E-12 |
| hsa-miR-4686 | 2.588876 | 4.05E-10 |
| hsa-miR-595 | 2.590481 | 4.35E-14 |
| hsa-miR-4436b-3p | 2.604608 | 7.65E-12 |
| hsa-miR-6739-3p | 2.613988 | 1.08E-13 |
| hsa-miR-3653-5p | 2.616104 | 9.46E-12 |
| hsa-miR-3685 | 2.62207 | 3.90E-13 |
| hsa-miR-4647 | 2.66596 | 4.73E-11 |
| hsa-miR-4773 | 2.687802 | 2.03E-10 |
| hsa-miR-892b | 2.76547 | 5.15E-13 |
| hsa-miR-4733-3p | 2.789818 | 9.52E-13 |
| hsa-miR-7850-5p | 2.878433 | 8.28E-15 |
| hsa-miR-3973 | 2.9009 | 1.33E-11 |
| hsa-miR-3650 | 2.906189 | 3.07E-14 |
| hsa-miR-487a-5p | 2.945489 | 1.33E-14 |
| hsa-miR-6801-5p | 2.994532 | 1.72E-14 |
| hsa-miR-4700-5p | 3.049286 | 3.00E-15 |
| hsa-miR-6736-5p | 3.192792 | 9.84E-18 |
| hsa-miR-4540 | 3.201044 | 8.19E-18 |
| hsa-miR-6072 | 3.215595 | 5.52E-17 |
| hsa-miR-6767-5p | 3.258755 | 1.86E-16 |
| hsa-miR-494-5p | 3.372208 | 2.45E-20 |
| hsa-miR-6740-5p | 3.548323 | 1.91E-21 |
| hsa-miR-6866-3p | 3.674317 | 2.29E-22 |
| hsa-miR-3064-5p | 3.968637 | 1.75E-24 |
| hsa-miR-4676-5p | 4.159822 | 1.93E-29 |

**Table S3. List of 356 DEmRNAs in the GSE58294 dataset**

| **DEmRNAs** | **log_2_Fold Change** | **adj. *p* value** |
| --- | --- | --- |
| ACSM2A | -3.174 | 1.08E-13 |
| POM121L9P | -3.14515 | 3.24E-16 |
| TIMM8A | -2.78234 | 1.08E-13 |
| SRCIN1 | -2.34228 | 3.95E-11 |
| DUXAP8 | -2.28368 | 1.43E-11 |
| SH3GL3 | -2.21257 | 5.52E-12 |
| LOC105377458 | -2.1308 | 5.47E-10 |
| LINC02076 | -2.08958 | 1.71E-13 |
| FAT3 | -2.0533 | 1.61E-09 |
| OVOL2 | -1.97832 | 1.16E-09 |
| GABRB2 | -1.92956 | 1.12E-08 |
| PRTG | -1.90869 | 2.34E-11 |
| FAM133A | -1.88963 | 7.73E-11 |
| GLOD5 | -1.84313 | 6.81E-07 |
| BTNL3 | -1.80913 | 0.010076 |
| ZNF536 | -1.80307 | 9.98E-11 |
| SPIB | -1.77543 | 3.65E-11 |
| GAGE1 | -1.75334 | 1.12E-08 |
| ANXA8 | -1.72229 | 3.68E-10 |
| DUBR | -1.69168 | 1.86E-06 |
| LECT2 | -1.67759 | 6.35E-08 |
| LPAR4 | -1.67131 | 3.96E-06 |
| THSD4 | -1.65716 | 1.78E-09 |
| ZNF133-AS1 | -1.65006 | 6.15E-10 |
| ZNF595 | -1.6468 | 0.000239 |
| LINC02249 | -1.64102 | 3.73E-09 |
| SIGLEC8 | -1.62767 | 2.43E-05 |
| PRSS33 | -1.61921 | 0.000549 |
| CCDC144CP | -1.6118 | 1.03E-10 |
| FAM102A | -1.60913 | 2.60E-13 |
| PPP5D1 | -1.58496 | 4.07E-09 |
| CD79B | -1.56391 | 4.19E-12 |
| RNF165 | -1.56306 | 1.92E-05 |
| RBMS3 | -1.53598 | 1.10E-08 |
| LOC441666 | -1.53486 | 2.12E-09 |
| SPTLC3 | -1.52536 | 1.28E-09 |
| LIX1 | -1.52419 | 2.00E-06 |
| MSX1 | -1.51117 | 5.80E-09 |
| ALDOAP2 | -1.48455 | 5.18E-10 |
| XRCC2 | -1.44157 | 1.59E-09 |
| FCRL4 | -1.43984 | 4.10E-09 |
| SHOX | -1.43408 | 2.31E-08 |
| ZNF727 | -1.42097 | 0.000658 |
| GADL1 | -1.41652 | 1.27E-06 |
| TNFRSF25 | -1.41365 | 6.80E-11 |
| COL5A3 | -1.40278 | 1.49E-06 |
| CD19 | -1.39833 | 1.29E-08 |
| RAD21-AS1 | -1.39602 | 2.91E-09 |
| TPH1 | -1.38416 | 0.00012 |
| ANKRD36B | -1.38301 | 1.10E-08 |
| PRO1804 | -1.37564 | 4.79E-07 |
| IGHD | -1.37039 | 6.30E-07 |
| NOG | -1.36226 | 1.50E-05 |
| NFIB | -1.35687 | 7.50E-05 |
| ZNF439 | -1.3546 | 2.77E-06 |
| ZNF594 | -1.34872 | 8.07E-07 |
| AGBL3 | -1.34711 | 0.000297 |
| CWF19L2 | -1.33661 | 7.25E-12 |
| ADAM23 | -1.33626 | 0.002204 |
| CXADR | -1.32838 | 5.18E-10 |
| TCL1A | -1.31528 | 6.38E-07 |
| LINC01798 | -1.30437 | 1.44E-06 |
| AKNAD1 | -1.30436 | 4.19E-05 |
| MPPED2 | -1.30175 | 6.21E-09 |
| CNTNAP2 | -1.29746 | 0.009306 |
| RASEF | -1.29124 | 2.47E-10 |
| EBF1 | -1.27489 | 4.27E-05 |
| CLIC3 | -1.2718 | 4.93E-08 |
| SPRY1 | -1.27074 | 2.34E-05 |
| RAG1 | -1.26836 | 3.23E-06 |
| CCR7 | -1.26819 | 5.35E-08 |
| LOC105372069 | -1.25747 | 6.19E-09 |
| UBE2S | -1.25643 | 7.12E-13 |
| GRIP1 | -1.25391 | 1.57E-05 |
| ZFP28 | -1.24976 | 4.32E-07 |
| LCN10 | -1.23894 | 5.41E-11 |
| LINC01527 | -1.23815 | 2.28E-05 |
| MYCT1 | -1.23474 | 7.29E-06 |
| LOC285097 | -1.23217 | 0.000538 |
| ZNF404 | -1.23199 | 0.00019 |
| SOX9 | -1.2306 | 1.04E-05 |
| TRAF7 | -1.23049 | 3.24E-16 |
| TPM2 | -1.22952 | 6.44E-10 |
| ACTR3BP2 | -1.21304 | 2.51E-07 |
| LRRC37BP1 | -1.20057 | 9.32E-06 |
| KLHL14 | -1.20053 | 0.000145 |
| ZNF781 | -1.19837 | 0.006913 |
| ARL6IP4 | -1.19806 | 4.65E-13 |
| ANKRD36BP2 | -1.19279 | 0.014819 |
| FAM155A | -1.18224 | 1.02E-07 |
| SNX22 | -1.1816 | 2.09E-08 |
| KCNH8 | -1.17884 | 0.000823 |
| FGF9 | -1.1771 | 0.000184 |
| ID3 | -1.17302 | 1.80E-10 |
| LOC93463 | -1.1662 | 2.70E-08 |
| LINC00624 | -1.16545 | 5.14E-07 |
| LOC105371215 | -1.16463 | 5.91E-06 |
| NEURL1 | -1.15764 | 1.79E-08 |
| SCN3A | -1.14921 | 0.008775 |
| ZNF711 | -1.14913 | 0.000239 |
| OLIG2 | -1.14816 | 0.003496 |
| PASK | -1.14753 | 1.06E-07 |
| UGT2B28 | -1.14213 | 1.90E-05 |
| UBE2G2 | -1.14191 | 1.81E-14 |
| C12orf42 | -1.13858 | 3.29E-06 |
| IDO1 | -1.13691 | 5.72E-05 |
| SYCP1 | -1.13593 | 3.49E-11 |
| CCL23 | -1.13568 | 0.012151 |
| BEX2 | -1.13464 | 1.98E-05 |
| PFDN6 | -1.13127 | 2.19E-13 |
| AOPEP | -1.12119 | 7.05E-08 |
| CLUHP3 | -1.12005 | 9.75E-09 |
| LOC100288656 | -1.11772 | 2.59E-05 |
| SPON1 | -1.11695 | 0.000162 |
| TMC6 | -1.11642 | 2.45E-12 |
| NELL2 | -1.11407 | 3.77E-06 |
| PDZK1IP1 | -1.11345 | 0.000125 |
| LOC100129917 | -1.11308 | 4.61E-06 |
| AFF3 | -1.11006 | 5.20E-06 |
| PMP2 | -1.1094 | 4.99E-05 |
| AXIN2 | -1.1086 | 2.05E-05 |
| LOC283788 | -1.10582 | 0.0024 |
| CD72 | -1.10467 | 1.37E-08 |
| VANGL2 | -1.10436 | 0.00095 |
| PAX8-AS1 | -1.09948 | 0.030093 |
| LINC00926 | -1.09938 | 9.98E-06 |
| DMRT2 | -1.09704 | 4.13E-06 |
| FREM3 | -1.09633 | 4.31E-05 |
| STRBP | -1.08664 | 1.15E-06 |
| NR1D2 | -1.08347 | 3.55E-09 |
| CELSR1 | -1.08313 | 0.000146 |
| TJP1 | -1.08211 | 0.000744 |
| OSBPL10 | -1.0806 | 9.28E-06 |
| IGHM | -1.08016 | 3.05E-06 |
| ALOX15 | -1.07996 | 0.003607 |
| SLC38A5 | -1.07691 | 3.20E-05 |
| FAM30A | -1.0738 | 1.31E-08 |
| FCRL5 | -1.06913 | 0.004635 |
| TRBV21-1 | -1.06746 | 4.08E-06 |
| CCDC144B | -1.06614 | 2.12E-05 |
| LINC00328 | -1.06498 | 0.000764 |
| CD27 | -1.0624 | 4.95E-08 |
| DRAP1 | -1.06239 | 1.71E-13 |
| IPO11 | -1.06062 | 0.000113 |
| LOC101927468 | -1.06055 | 7.31E-06 |
| GNAZ | -1.0603 | 0.000178 |
| FAM153B | -1.0564 | 5.85E-05 |
| TUB | -1.05623 | 4.43E-07 |
| LOC100505915 | -1.0553 | 6.85E-08 |
| TSGA10 | -1.05482 | 0.021123 |
| LINC00662 | -1.04891 | 2.56E-06 |
| SEPSECS-AS1 | -1.0438 | 1.16E-08 |
| LOC202181 | -1.04293 | 3.11E-05 |
| LTB | -1.03982 | 7.14E-12 |
| CBR3 | -1.03934 | 0.000124 |
| COL4A3 | -1.0359 | 0.00238 |
| FCRL1 | -1.03445 | 0.000405 |
| ISM1 | -1.03377 | 0.000976 |
| MRPL41 | -1.03355 | 1.54E-10 |
| TMEM161B-AS1 | -1.03338 | 5.65E-09 |
| SMIM24 | -1.03332 | 0.009433 |
| KLHL13 | -1.03325 | 0.000138 |
| FAM111B | -1.02982 | 1.20E-05 |
| MRPL12 | -1.0286 | 1.04E-10 |
| LINC01184 | -1.02838 | 3.02E-07 |
| TAF3 | -1.02758 | 8.89E-09 |
| ZNF415 | -1.02757 | 0.007932 |
| MMP19 | -1.02485 | 1.63E-11 |
| P2RX5 | -1.02328 | 1.10E-07 |
| GAS6-AS1 | -1.02075 | 0.00067 |
| ATXN2L | -1.01801 | 4.20E-10 |
| ZFP14 | -1.01787 | 0.000925 |
| PTPRD | -1.01773 | 0.000552 |
| SEPTIN1 | -1.01727 | 1.08E-08 |
| SLC9A3-AS1 | -1.01594 | 4.14E-06 |
| UICLM | -1.0144 | 0.00017 |
| PDE9A | -1.0135 | 0.000156 |
| FAM174C | -1.01067 | 3.49E-11 |
| SIGLEC6 | -1.01053 | 0.001098 |
| APBA2 | -1.01041 | 1.11E-08 |
| TRAV23DV6 | -1.01026 | 0.001423 |
| LINC02754 | -1.00962 | 7.12E-05 |
| PIEZO1 | -1.0096 | 1.37E-11 |
| PROM1 | -1.00887 | 0.003851 |
| ACKR4 | -1.00787 | 1.73E-05 |
| ZNF570 | -1.00645 | 1.23E-05 |
| CCEPR | -1.00234 | 7.09E-06 |
| ZNF521 | -1.00222 | 2.80E-08 |
| NT5E | -1.00166 | 7.27E-05 |
| RASA4 | -1.00078 | 8.15E-08 |
| PTP4A3 | -1 | 3.75E-07 |
| ABHD5 | 1.00092 | 5.41E-08 |
| SLC5A9 | 1.003047 | 0.00054 |
| CDCA5 | 1.003585 | 1.81E-06 |
| LYVE1 | 1.005132 | 0.000306 |
| PLXDC2 | 1.005516 | 4.70E-10 |
| SFXN5 | 1.005545 | 3.40E-07 |
| ZNF438 | 1.008092 | 4.89E-08 |
| MPZL2 | 1.009621 | 1.77E-05 |
| TLR5 | 1.011358 | 4.18E-07 |
| LOC114224 | 1.012249 | 3.16E-06 |
| RPH3A | 1.014215 | 0.008222 |
| CFAP58 | 1.01598 | 8.77E-06 |
| TCN1 | 1.016683 | 0.000176 |
| SERTAD3 | 1.017084 | 5.47E-08 |
| PGLYRP1 | 1.017362 | 6.98E-06 |
| TENM1 | 1.018225 | 0.001245 |
| SBNO2 | 1.022873 | 3.41E-09 |
| TANC2 | 1.026228 | 1.79E-09 |
| ZNF818P | 1.027115 | 0.002232 |
| NSUN7 | 1.027392 | 2.63E-05 |
| OLR1 | 1.03516 | 0.030508 |
| LOC100506100 | 1.035194 | 8.79E-09 |
| ITGA1 | 1.035376 | 3.15E-07 |
| SLC37A3 | 1.039521 | 5.29E-07 |
| SEMG1 | 1.041712 | 0.003999 |
| BFSP2-AS1 | 1.042414 | 0.001916 |
| DNAI7 | 1.043068 | 4.98E-06 |
| GPR141 | 1.049676 | 0.000111 |
| GAB1 | 1.050246 | 6.19E-09 |
| KIAA0825 | 1.051437 | 2.08E-07 |
| KREMEN1 | 1.051763 | 3.18E-05 |
| DPF2 | 1.0554 | 7.79E-10 |
| SPRED2 | 1.057012 | 4.57E-07 |
| TGFBR3 | 1.059764 | 5.95E-05 |
| TRIM9 | 1.062594 | 0.003076 |
| SLC22A4 | 1.067618 | 5.86E-10 |
| DSC2 | 1.067806 | 4.13E-05 |
| MS4A4A | 1.072123 | 7.41E-05 |
| EFNA5 | 1.074538 | 1.78E-05 |
| LINC02656 | 1.075074 | 4.61E-06 |
| ANXA3 | 1.078384 | 8.73E-05 |
| COX7B | 1.079812 | 4.73E-05 |
| C1orf226 | 1.081494 | 6.18E-07 |
| TLCD4 | 1.08434 | 0.024024 |
| ACOX2 | 1.084619 | 9.47E-08 |
| RAB36 | 1.086229 | 5.33E-08 |
| GK3P | 1.086485 | 3.30E-10 |
| LOC101927851 | 1.090286 | 2.06E-06 |
| LINC00671 | 1.09314 | 2.90E-06 |
| NME8 | 1.09481 | 1.22E-07 |
| RPL22L1 | 1.095561 | 6.89E-05 |
| CHRNA10 | 1.097129 | 8.50E-10 |
| SIPA1L2 | 1.099517 | 5.85E-07 |
| HEPACAM2 | 1.099825 | 0.042074 |
| H4C8 | 1.101858 | 7.04E-07 |
| CASTOR3 | 1.10293 | 2.25E-08 |
| BTBD19 | 1.103262 | 1.09E-06 |
| TMEM45B | 1.105018 | 5.53E-05 |
| IL18RAP | 1.105141 | 1.11E-08 |
| FOLR3 | 1.107416 | 0.002062 |
| LOC100130744 | 1.108278 | 5.43E-08 |
| CILP | 1.112958 | 0.000315 |
| ANXA9 | 1.115766 | 2.82E-06 |
| OSBPL1A | 1.116589 | 1.38E-07 |
| FAR2 | 1.120402 | 2.56E-09 |
| ERVK3-2 | 1.122777 | 0.000932 |
| MIER2 | 1.125498 | 1.89E-09 |
| F5 | 1.128601 | 2.39E-09 |
| GPR84 | 1.129668 | 0.00013 |
| BCL2A1 | 1.131915 | 9.62E-07 |
| OMG | 1.132803 | 0.000794 |
| SNORD89 | 1.134825 | 3.31E-07 |
| TGFA | 1.135978 | 2.39E-07 |
| GLIS2 | 1.138856 | 1.62E-06 |
| ASPH | 1.141681 | 4.94E-07 |
| AK4 | 1.142009 | 7.88E-07 |
| LINC01093 | 1.142096 | 0.003155 |
| BCAT1 | 1.144861 | 2.01E-05 |
| CFD | 1.156594 | 0.000111 |
| SAP30 | 1.157763 | 1.16E-07 |
| OSM | 1.157816 | 2.23E-07 |
| DDIT4L | 1.15942 | 3.57E-06 |
| SPAG6 | 1.16215 | 5.36E-05 |
| SHOC1 | 1.164972 | 9.87E-06 |
| CLEC5A | 1.165937 | 1.80E-06 |
| NSG2 | 1.167948 | 0.000129 |
| SLC22A1 | 1.169047 | 1.58E-05 |
| ZNF608 | 1.176655 | 0.001445 |
| ZNF467 | 1.177115 | 5.71E-09 |
| PRDM5 | 1.182553 | 0.000454 |
| H2AC15 | 1.182964 | 0.00018 |
| KL | 1.184692 | 0.004894 |
| LINC00862 | 1.190821 | 3.75E-06 |
| S1PR3 | 1.196439 | 7.79E-07 |
| FKBP9 | 1.203303 | 1.87E-07 |
| MIR3945HG | 1.203712 | 8.31E-05 |
| C3orf62 | 1.205878 | 9.53E-11 |
| HECW2 | 1.212447 | 2.61E-08 |
| KCNH7 | 1.215164 | 3.34E-08 |
| F12 | 1.216343 | 1.13E-11 |
| ECHDC3 | 1.216867 | 0.000485 |
| LOC101929305 | 1.219715 | 2.45E-05 |
| CYP1B1-AS1 | 1.224214 | 0.00123 |
| TMEM272 | 1.225058 | 1.16E-08 |
| GSEC | 1.226179 | 2.39E-07 |
| CNTNAP3P2 | 1.228191 | 0.008767 |
| CHPF2 | 1.235756 | 8.08E-13 |
| OR2B6 | 1.238432 | 2.98E-06 |
| RGL3 | 1.239578 | 3.53E-08 |
| PLIN5 | 1.240943 | 8.71E-08 |
| DRC1 | 1.241643 | 2.89E-06 |
| SCRG1 | 1.242654 | 4.51E-08 |
| CASP5 | 1.252863 | 3.04E-06 |
| C1QC | 1.255212 | 1.29E-06 |
| SYN2 | 1.257852 | 7.33E-05 |
| LLCFC1 | 1.258854 | 3.26E-06 |
| IL1R1 | 1.262742 | 7.66E-08 |
| ITCH | 1.266775 | 2.62E-06 |
| RSPH14 | 1.273546 | 1.79E-08 |
| ABCA1 | 1.275162 | 1.81E-06 |
| ADGRG3 | 1.277024 | 9.78E-10 |
| HNRNPL | 1.281224 | 2.06E-09 |
| PNPLA1 | 1.283182 | 1.23E-06 |
| C8orf88 | 1.291373 | 1.70E-07 |
| KAZN | 1.291827 | 0.002361 |
| GJB6 | 1.294655 | 0.001138 |
| CCDC71L | 1.295268 | 9.41E-10 |
| LOC100422526 | 1.295502 | 1.07E-06 |
| FRY-AS1 | 1.295657 | 1.95E-06 |
| METTL7B | 1.334354 | 0.00671 |
| TPST1 | 1.350405 | 1.06E-05 |
| PRL | 1.355375 | 0.000107 |
| LOC105375769 | 1.361289 | 2.07E-06 |
| GALNT14 | 1.369824 | 3.11E-07 |
| ST6GALNAC3 | 1.379574 | 5.63E-09 |
| MTARC1 | 1.393455 | 7.41E-09 |
| LINC01270 | 1.407966 | 7.86E-05 |
| TTN | 1.415027 | 5.86E-10 |
| BTNL8 | 1.418195 | 0.033402 |
| MOCS1 | 1.428767 | 0.001443 |
| DLGAP1-AS2 | 1.430192 | 1.01E-11 |
| LRRN1 | 1.432749 | 1.07E-05 |
| LOC101928236 | 1.441551 | 5.69E-07 |
| PRRG4 | 1.443928 | 5.33E-10 |
| SMIM27 | 1.455328 | 2.91E-13 |
| HTRA1 | 1.519765 | 7.97E-06 |
| MMP9 | 1.520752 | 1.15E-06 |
| CLEC4D | 1.547698 | 1.18E-09 |
| H4C4 | 1.568441 | 3.45E-09 |
| ANKRD22 | 1.594654 | 3.96E-06 |
| LINC02555 | 1.620112 | 4.98E-07 |
| CD177 | 1.648475 | 0.002971 |
| SLC26A8 | 1.690266 | 6.28E-08 |
| LILRA5 | 1.707051 | 4.96E-11 |
| MCEMP1 | 1.751564 | 1.64E-09 |
| DAAM2 | 1.821297 | 0.001102 |
| BMX | 1.823598 | 1.12E-08 |
| INSC | 1.907569 | 7.70E-08 |
| VSIG4 | 1.927156 | 6.40E-08 |
| MAOA | 1.93608 | 0.000552 |
| FCAR | 1.95709 | 9.56E-09 |
| CACNA1E | 1.984141 | 4.62E-09 |
| FGF13 | 1.987185 | 0.000831 |
| ARG1 | 2.400864 | 2.13E-08 |
| OLAH | 2.939408 | 2.90E-09 |
